# Meta-Analysis of Goal Setting and Physical Treatment Categorisation for Focal Spasticity Following Stroke or Other Acquired Brain Injury

**DOI:** 10.1177/27536351251343520

**Published:** 2025-06-19

**Authors:** Stephen Ashford, Jorge Jacinto, Klemens Fheodoroff, Lynne Turner-Stokes

**Affiliations:** 1 Department of Palliative Care, Policy and Rehabilitation, Cicely Saunders Institute, Florence Nightingale Faculty of Nursing, Midwifery and Palliative Care, King’s College London, UK; 2 Regional Hyper-Acute Rehabilitation Unit, Northwick Park Hospital, London North West University Healthcare, UK; 3 Centro de Medicina de Reabilitaçãode Alcoitão, Serviço de Reabilitação de adultos 3, Estoril, Portugal; 4 Department of Neurorehabilitation, Gailtal-Clinic, Hermagor, Austria

**Keywords:** botulinum toxin A, goal attainment scaling, physical therapies, post-stroke spasticity, stroke rehabilitation

## Abstract

**Background:**

Setting goals and planning treatment to attain those goals is often integral to rehabilitation practice, particularly when managing spasticity following stroke or other brain injury. Optimal treatment planning and provision using an algorithm based on mapping goals and treatments, may improve outcome.

**Methods:**

We analysed goal setting and treatment interventions through secondary analysis of goals and related treatments from (a) the Leg Activity measure study, (b) Ankle Contracture data set and (c) the Upper Limb International Spasticity-III study. Total 1207 participants. Goal categories were defined and identified based on a previously published framework: Pain, Involuntary Movement, Contracture Prevention, Active Function (self-performance of tasks), passive function (secondary performance of tasks or personal care). Treatment intervention was then identified per goal category.

**Results:**

*Arm spasticity goal categorisation*: Pain 302 (22%), Involuntary Movement 166 (12%), Contracture Prevention 208 (15%), Active Function 174 (13%), passive function 501 (37%). *Primary interventions identified per category*: Pain (Positioning the limb, serial casting), Involuntary Movement (Position the limb, Splinting), Contracture Prevention (Positioning the limb, serial casting, Shoulder support and slings, Splinting), Active Function (Positioning the limb, serial casting, shoulder supports and splinting), passive function (Positioning the limb, serial casting, shoulder supports and splinting). *Leg spasticity goal categorisation*: Pain 117 (15%), Involuntary Movement 10 (1%), Contracture Prevention 139 (17%), Active Function 356 (44%), passive function 181 (22%). *Primary interventions identified per category*: Pain (Passive stretch, positioning), Involuntary Movement (Splinting), Contracture Prevention (Positioning, Orthotics, Task Practice), Active Function (Task Practice, Orthotics), passive function (Orthotics, Positioning).

**Conclusions:**

Commonalities in goal categorisation were found in arm and leg. In these cohorts’ task-practice interventions to improve active function (walking and transferring) were reported for leg but were not frequently reported for arm rehabilitation. It is suggested that improved treatment planning may result in greater and faster treatment goal attainment and better outcomes.

## Introduction

Many people have difficulty controlling muscles following stroke and brain injury. Some are also left with muscle tightness called ‘spasticity’. Spasticity is often painful, like muscle cramp. It can limit mobility and independence and contribute to changes in muscle structure leading to contractures, skin damage or pressure sores. Information on the number of people with spasticity is varied, but it has been reported in up to 42% of stroke survivors (approximately 504 000 people in the UK).^1-3^ Spasticity is a disabling problem limiting many functional activities and rehabilitation progress, but there are solutions and ways to control it.

National clinical guidelines further advised that physical management is fundamental to treatment and BoNT-A injections are an adjunct to meeting the wider rehabilitation aims of the patient when spasticity is present. Spasticity management is well defined for pharmacological treatment (eg, Botulinum toxin), but how it is best combined with the whole rehabilitation programme is not as clear.^4^ Although this clinical guideline explicitly states, ‘spasticity management should be part of a goal-oriented programme, centred on the patient’s priority goals for treatment’, nationally (and internationally), spasticity treatment is not always well linked to the rehabilitation goals of people following stroke and brain injury.

The UK and Ireland National Clinical Guidelines for Stroke^5^ recommend that multidisciplinary teams should incorporate practice of functional skills gained in therapy into the person’s daily routine in a consistent manner, and the care environment should support people with stroke to practise their activities as much as possible (Study Participants Section). Functional activities should be individualised to the person’s goals and interests. Ensuring individualised goal directed treatment planning is therefore widely acknowledged as important and is particularly needed for those with spasticity who often have more severe disability.

A physical treatment recording system was developed and then applied in the Upper Limb International Spasticity study III (ULIS-III) which was a longitudinal international programme following 1004 people with arm spasticity following stroke across 84 centres in 14 countries.^6^ The Upper Limb International Spasticity (ULIS) study programme incorporates a series of observational studies designed to describe real-life clinical practice in the use of BoNT-A to manage arm spasticity.^7^ All participants included had arm spasticity and were able to consent to treatment, participants lacking capacity to consent, often with more severe disability, were excluded in these cohorts.

The first 2 studies (ULIS-I and ULIS-II^7,8^) documented current practice and confirmed the feasibility of a common international dataset to collect prospective data incorporating Goal Attainment Scaling (GAS). The dataset captures outcomes across a range of goal areas that included passive and active function subsequently incorporating the Arm Activity (ArmA) measure,^9,10^ as well as pain and mobility. The third study (ULIS-III)^11^ described the effects of integrated spasticity management with repeated cycles of BoNT-A over 2 years. It introduced novel methods to (i) systematically capture integrated approaches to spasticity management, including multidisciplinary therapy inputs using the Upper Limb Therapy Recording Schedule (ULSTR) and (ii) the Upper Limb Spasticity Index (ULSI),^4^ which combines GAS with targeted standardised measures such as the ArmA (selected according to the patient’s priority goals for treatment).

Similar work has been undertaken for leg spasticity management with the development and then subsequent testing of the Leg Activity measure (LegA).^12^ The studies using the LegA have also incorporated methods to (i) systematically capture integrated approaches to spasticity management in the leg, including multidisciplinary therapy inputs, using the Leg Therapy recording Schedule (LegTS)^13^ and (ii) systematic goal setting and attainment^14^ (Royal College of Physicians, British Society of Rehabilitation Medicine et al. 2018) and leg function using the Leg Activity measure.^15^

## Goal Classification Approach

In an earlier analysis,^16^ we developed a goal classification of individualised goals for spasticity treatment (incorporating botulinum toxin intervention) for upper limb spasticity to under-pin a more structured approach to future goal setting.

Individualised goals for spasticity treatment incorporating botulinum toxin intervention for upper limb spasticity (n = 696) were analysed initially from 4 studies published in 2008 to 2012, spanning a total of 18 centres (12 in the UK and 6 in Australia).^8,17-19^ Goals were categorised and mapped onto the closest matching domains of the WHO International Classification of Functioning, disability and health.

Goal categories could be assigned into 2 domains, each subdivided into 3 key goal areas:

Domain 1: symptoms/impairment n = 322 (46%): (a) pain/discomfort n = 78 (11%), (b) involuntary movements n = 75 (11%) and (c) range of movement/contracture prevention n = 162 (23%).
Domain 2: Activities/function n = 374 (54%): (a) passive function (ease of caring for the affected limb) n = 242 (35%), (b) active function (using the affected limb in active tasks) n = 84 (12%) and (c) mobility n = 11 (2%).

Confirmatory analysis included a further 927 goals from a large international cohort study spanning 22 countries published in 2013.^8^ Over 99% of the goals from the large international cohort fell into the same 6 areas, confirming the consistency and international applicability of the classification.^20^

The aim of the current meta-analysis was therefore to use the goal categorisation system developed,^16^ to initially categorise goals set under the pre-defined categories. Following this, the goals in each category would be used to identify therapy intervention given to better understand what physical treatment was applied (in addition to botulinum toxin) in each pre-identified goal category. It was expected that treatment goal would have a substantial influence on the type of treatment required.

It is anticipated that the outcomes from this analysis will inform a physical treatment planning algorithm to better aid targeting of treatment and become a standard for practice to enable improved and optimal rehabilitation for stroke survivors and others with spasticity. The algorithm will incorporate new treatment interventions and approaches as they emerge. It will also incorporate different treatment settings, such as in-patient or community-based and in particular support individualised task-training or self-guided rehabilitation. It is suggested that improved treatment targeting may result in greater and faster treatment goal attainment and better outcome for stroke survivors and others following acquired brain injury.

## Research Questions

1. RQ1: Using the pre-defined goal framework for spasticity management, what types of physical treatment intervention are provided according to goal category in the arm and the leg in people following an acquired brain injury (including stroke).
2. RQ2: What proportions of physical intervention type are provided in each goal category (Pain, Involuntary Movement, Contracture Prevention, Active Function and Passive function).

## Methods

### Included Studies

Included studies were selected on the basis of capturing systematic data on goals for treatment using a structured approach to goal attainment scaling (Goal Attainment Scaling Evaluation of Outcome for Upper Limb Spasticity (GAS-eous) tool,^21^ or Goal Attainment Scaling for treatment of leg spasticity (GAS-Legs) tool^14^ and use of systematic approaches to capturing physical treatment interventions using a standardised method, such as the Upper Limb Spasticity Therapy Recording schedule (ULSTR)^11^ or the Leg-Therapy recording Schedule (Leg-TS).^13^

On this basis the following studies were selected based on applying these measures and processes: ULIS-III study,^6^ the Leg Activity measure (LegA) psychometric study^15^ and the Ankle Contracture cohort.^22^

### Design

The secondary meta-analysis of goal setting was undertaken to identify goal category based on the previous categorisation model developed.^14,21^ Goal statements were extracted, classified to determine goal categories in phase I. Classification of goals was completed by the first author (SA) independently. For classification, goals were grouped to identify the categories from the datasets.

In phase II, treatment intervention data from (a) ULIS-III study,^6^ (b) the Leg Activity measure (LegA) psychometric study^15^ and the (c) Ankle Contracture cohort^22^ were identified linked to the goal category.

Standard reporting frameworks were considered in presenting this work in the form of the MOOSE Reporting guidelines for meta-analyses of observation studies.^23^ The MOOSE checklist has not been applied, because a systematic review with literature searching (to which the checklist relates) was not completed as part of this study.

### Study Participants

Study participants were recruited and followed up in the 3 studies as follows:

### Upper Limb International Spasticity III Study (ULIS-III)

In ULIS III, a total of 1004 participants were enrolled across the 58 active centres, of which 990 entered Cycle 1, and 953 underwent ⩾1 BoNT-A injection cycle and had ⩾1 GAS assessment and were included in the effectiveness population. All BoNT-A injections administered within the 2-year period were captured as a cycle of treatment, alongside the primary elements of the rehabilitation programme for up to 9 cycles. The main reasons for premature study withdrawal were logistics (eg, ability to attend visits) and loss to follow-up.

### Leg Activity Measure Study (LegA-S)

A prospective consecutive cohort of participants (n = 64) with lower limb spasticity presenting for treatment at 1 of 3 specialist rehabilitation services in UK. Inclusion in the cohort study was based on a clinically identified need for spasticity management (including botulinum toxin injection) as part of a rehabilitation programme. Services were chosen purposively to reflect the range/types of specialist spasticity services available in the UK, but with relevance more widely to other countries and health systems.

### Ankle Contracture Cohort (Ankle-SC)

The Ankle-SC cohort was a prospective consecutive cohort of participants (n = 101) from a single specialist rehabilitation service in the UK. Participants with acquired brain injury (primarily stroke), received customised inpatient rehabilitation programmes based on clinical need as decided by the therapy clinicians working with them including management of spasticity in the leg. Prior to clinical treatment participants were fully assessed for both active and passive range of movement, sensation, motor-control (active ankle dorsiflexion), spasticity and functional performance. Assessment findings were considered in delivery of the rehabilitation programmes provided, with issues such as spasticity addressed. Of the 357 people assessed, 101 (28%) individuals were identified as having an ankle contracture (and spasticity) were included.

### Measurement

#### Goal Attainment Scaling

Goals for treatment were captured using the Goal Attainment Scaling Evaluation of Outcome for Upper Limb Spasticity (GAS-eous) tool,^21^ or Goal Attainment Scaling for treatment of leg spasticity (GAS-Legs) tool^14^ which apply a structured approach to goal-setting and evaluation of goal attainment which is assimilated to yield a GAS T score utilising the GAS light method.^17,21^

If goals are achieved as expected, the mean GAS *T* score would be 50. The minimal clinically important change in GAS T score from baseline is reported to be 10.^21^ Training and proactive feedback on goals set from an early stage in the ULIS-III and LegA-S studies helped to ensure high-quality goal setting process and further supported the validity of GAS as a measure of achievement of the intended goals for treatment.^21^

#### Upper Limb Spasticity Therapy Recording Schedule (ULSTR)

In ULIS III, physical therapy intervention was captured using the *Upper Limb Spasticity Therapy Recording schedule (ULSTR)* developed by Ashford and Turner-Stokes.^11^ The ULSTR recording Schedule was developed to specifically capture physical therapy intervention provided alongside pharmacological management for spasticity in the context of a rehabilitation programme. The ULSTR has 2 sections, both capturing type of therapy interventions. Section A captures intervention ranging in dose/time from less than an hour/per day to greater than 6 hours/per day. Section B captures intervention ranging in dose/time from less than 15 minutes per day to greater than 1 hour per day. The difference between the interventions in the different sections is purely based on the likely time duration for which they are provided.

#### Leg Therapy Schedule

Therapy intervention was captured using the *Leg-Therapy recording Schedule*. The Leg-TS recording Schedule was developed to specifically capture therapy intervention provided alongside pharmacological management for spasticity in the context of a rehabilitation programme.^13^ The Leg-Therapy recording Schedule has 2 sections, both capturing type of therapy interventions. Section A captures intervention ranging in dose/time from less than an hour/per day to greater than 6 hours/per day. Section B captures intervention ranging in dose/time from less than 15 minutes per day to greater than 1 hour per day. The difference between the interventions in the different sections is purely based on the time duration for which they are provided.

### Ethical Approval

Data used in the analysis were de-identified. Ethical approval, though technically not required for secondary data analysis was provided following review by the UK Heath Research Authority (REC 22/HRA/2869).

## Results

### Participant Baseline Characteristics

Baseline characteristics are provided in Table 1 for each of the studies. Over the 3 studies, 56% of patients were male and the mean ± SD age was 48.6 ± 15 years. On average, participants were living with chronic spasticity. For the large majority of participants, spasticity occurrence was secondary to a vascular event (stroke).

**Table 1.** Baseline demographics and severity of presentation.

Parameter	Arm – ULIS III (N = 953)	LegA (N = 64)	Leg ankle (N = 101)
Age (years); mean (SD)	54.0 (15.3)	51.0 (17.4)	40.9 (12.25)
Sex; n (%) male/female	537:416 (56/44%)	32:32 (50/50%)	57:44 (56/44%)
Time since onset of the event leading to spasticity Mean (SD)	7.6 years (9.4)	–^a^	117 days (64.51)
Acquired brain injury (stroke/trauma/other)	870 (91.3%)	49 (76%)	85 (84%)
Spinal cord injury	15 (1.6%)	1 (1%)	0 (0%)
Progressive neurological condition	20 (2.1%)	8 (13%)	10 (10%)
Congenital	44 (4.6%)	6 (9%)	4 (4%)
Other	4 (0.4%)	–^a^	(2%)

a Data not available.

### Spasticity Management

All participants (1118; 100%) in the included data sets underwent spasticity management including physical rehabilitation and, in most cases, pharmacological treatment for spasticity with botulinum toxin injection (1028; 92%).

### Change Following Intervention

Full outcome and change findings from the 3 included cohorts are not reported in this analysis. Primary outcome from intervention is summarised below with reference to the relevant publications for further information.

In the ULIS III cohort, goal-setting and goal-attainment assessment were implemented using the GASeous tool.^21^ Overall goal attainment of the 828 treated patients with GAS T-score response data, 71.9% of patients (n = 595) had a 10-point or greater improvement in GAS *T* score from baseline to follow-up for each cycle (denoted by botulinum toxin administration), with a cumulated mean GAS T-score (95% CI) of 49.5 (49.1, 49.9).^11^ Up to 9 cycles of treatment were delivered dependent on clinical need. For full outcome data please see the full publication of longitudinal data.^11^

In the Leg-AS cohort, participants received a single cycle of treatment (denoted by botulinum toxin administration), with a median GAS T-score of 50 (42.5-50).

In the Ankle-AS cohort, categorisation was undertaken according to the degree of reduced range of movement at the ankle joint, mild(>plantigrade), moderate(<plantigrade to -20°) or severe(>-21°). Significant change in range of movement was not seen in the mild category (1.05; P = .29), but was in the moderate (5.436; *P* = .001) and severe categories (10.4; *P* = .001). Improvement in locomotion was noted in all 3 groups (6.10; *P* = .001) but was most pronounced in the moderate and severe groups.^22^

### Goal and Physical Therapy Categorisation

#### Arm Spasticity Goal Categorisation

In the ULIS-III cohort the goals for arm spasticity treatment were categorised under the pre-identified domains as follows:

- Pain 302 (22%),
- Involuntary Movement 166 (12%),
- Contracture Prevention 208 (15%),
- Active Function 174 (13%),
- Passive function 501 (37%).

#### Primary Physical Interventions Identified Per Goal Category

The primary interventions identified were.

- Pain: Positioning the limb, serial casting.
- Involuntary Movement: Position the limb, Splinting.
- Contracture Prevention: Positioning the limb, serial casting, shoulder support and slings, Splinting.
- Active Function: Positioning the limb, serial casting, shoulder supports and splinting.
- Passive function: Positioning the limb, serial casting, shoulder supports and splinting.

Arm physical Intervention by goal category for 4 cycles of treatment (ULIS III, n = 953) are presented in Table 2.

**Table 2.** Arm physical intervention by goal category for 4 cycles of treatment (upper limb international spasticity study III, n = 953).

Physical interventions	Cycle 1	Cycle 2	Cycle 3	Cycle 4	Total (percentage)
Active function (goal category)	58	48	38	30	174 (13)
Positioning of upper limb	37	29	23	17	108 (62)
Passive stretch	35	28	22	16	103
Strength training	1				1
Task practice	1	1	1	1	4
Serial casting	2	2			4 (2)
Passive stretch	2	2			4
Shoulder supports/slings	1				1 (1)
Passive stretch	1				1
Splinting	18	17	15	13	63 (36)
Passive stretch	17	16	14	12	59
Strength training	1	1	1	1	4
Involuntary movements (goal category)	56	49	38	23	166 (12)
Positioning of upper limb	30	25	20	10	85 (51)
Passive stretch	27	22	17	10	76
Task practice	3	3	3		9
Splinting	26	24	18	13	**81 (49)**
Passive stretch	26	24	18	13	81
Pain (goal category)	104	85	70	43	302 (22)
Positioning of upper limb	47	37	25	16	125 (41)
Passive stretch	44	34	23	14	115
Strength training	3	3	2	2	10
Serial casting	2	2	2	1	7 (2)
Passive stretch	2	2	2	1	7
Shoulder supports/slings	8	7	7	4	26 (9)
Passive stretch	8	7	7	4	26
Splinting	47	39	36	22	144 (48)
Electrical stimulation	1	1	1	1	4
Passive stretch	45	37	34	20	136
Task practice	1	1	1	1	4
Passive function (goal category)	156	145	123	77	501 (37)
Positioning of upper limb	84	80	65	42	271 (54)
Electrical stimulation	1	1	1	1	4
Passive stretch	81	77	62	40	260
Task practice	2	2	2	1	7
Serial casting	2				2 (0)
Passive stretch	2				2
Shoulder supports/slings	8	8	8	2	26 (5)
Passive stretch	8	8	8	2	26
Splinting	62	57	50	33	202 (40)
Electrical stimulation	1	1	1		3
Passive stretch	60	55	49	33	197
Strength training	1	1			2
Contracture prevention (goal category)	67	62	46	33	208 (15)
Positioning of upper limb	39	37	30	19	125 (60)
Electrical stimulation	1	1	1	1	4
Passive stretch	36	34	27	17	114
Strength training	1	1	1	1	4
Task practice	1	1	1		3
Serial casting	2	2			4 (2)
Passive stretch	2	2			4
Shoulder supports/slings	1				1 (0)
Passive stretch	1				1
Splinting	25	23	16	14	78 (38)
Electrical stimulation	1	1	1	1	4
Passive stretch	24	22	15	13	74
Grand total	442	390	315	206	1353 (100)

#### Leg Spasticity Goal Categorisation

In the LegA-S and Ankle-SC cohorts the leg goals were categorised under the pre-identified domains as follows:

- Pain 117 (15%),
- Involuntary Movement 10 (1%),
- Contracture Prevention 139 (17%),
- Active Function 356 (44%),
- Passive function 181 (22%).

#### Primary Interventions Identified Per Category

The primary interventions identified were.

- Pain: Passive stretch, positioning.
- Involuntary Movement: Splinting.
- Contracture Prevention: Positioning, Orthotics, Task Practice.
- Active Function: Task Practice, Orthotics.
- Passive function: Orthotics, Positioning.

Leg physical Intervention by goal category for (LegA-S and Ankle-S, n = 803) are presented in Table 3.

**Table 3.** Leg physical intervention by goal category for 1 cycle of treatment (leg activity measure validation study [n = 64]; Ankle contracture cohort study [n = 190]).

Goal category	Splinting	Orthotics	Task practice	Standing programme	Serial Casting	Strength training	Electrical Stimulation	Passive stretch	Positioning	Total
Pain	20	15	7	5	0	9	0	30	31	117 (15%)
Involuntary movement (Spasm)	5	0	3	0	0	0	2	0	0	10 (1%)
Prevention of Contracture (Range of movement)	20	25	25	1	1	5	7	22	33	139 (17%)
Passive Function	14	8	21	8	0	21	6	36	67	181 (23%)
Active Function (Locomotion or Transfers)	11	76	110	56	21	18	8	30	26	356 (44%)
Total	70 (9%)	124 (15%)	166 (21%)	70 (9%)	22 (3%)	53 (7%)	23 (3%)	118 (15%)	157 (20%)	803 (100%)

## Discussion

This is the first meta-analysis study to our knowledge, which captures the physical treatments given for rehabilitation in the context of spasticity for both the arm and leg. Commonalities in goal categorisation were found in arm and leg rehabilitation in individuals who also have spasticity. Differences in the proportion of goals set do however occur. Importantly, in these cohorts’ task-practice interventions to improve active function (walking and transferring) were applied to rehabilitation for leg spasticity but were not frequently applied in arm rehabilitation (eg, object use, incorporation in activities of daily living task practice) for those with spasticity.

### Treatment Categorisation Based on GOAL Category

For arm spasticity the primary interventions applied per category by goal heading were: Pain (Positioning the limb, serial casting), Involuntary Movement (Position the limb, Splinting), Contracture Prevention (Positioning the limb, serial casting, Shoulder support and slings, Splinting), Active Function (Positioning the limb, serial casting, shoulder supports and splinting) and passive function (Positioning the limb, serial casting, shoulder supports and splinting). Strength training‚ task practice and electrical stimulation were not frequently applied, which is surprising given that these interventions have more extensive evidential support for application in active function improvement. Task practice intervention is complex, and programmes have been developed to facilitate delivery further such as Constraint Induced Movement Therapy^24^ or the GRASP programme.^25^ Application of these programmes was however not seen in this cohort. This raises the concern that targeted and evidenced based interventions may not be being applied to task training for those with arm spasticity who still have active function goals.

For leg spasticity the primary interventions applied per category by goal heading were: Pain (Passive stretch, positioning), Involuntary Movement (Splinting), Contracture Prevention (Positioning, Orthotics, Task Practice), Active Function (Task Practice, Orthotics), passive function (Orthotics, Positioning). Task practice or training was more frequently applied in the leg cohort, which may reflect the generally higher functional ability of this group (ie, able to walk 5 steps or more for inclusion into the study for the Abolish cohort). Strength training and electrical stimulation were applied, but not as frequently as might be expected.

The lack of task specific training applied in rehabilitation of the arm (compared to leg) for those with spasticity in this secondary analysis may relate in part to the severity of paresis and more limited motor control. In the arm, when compared with the leg, greater motor control is required in general for functional activity performance. In a recent study of leg spasticity treatment called Abolish^26,27^ there was a requirement that participants be able to walk 5 steps or more and were therefore more likely to engage in functional rehabilitation (active function). The leg spasticity groups in this study were more impaired generally and many could not have fulfilled this requirement. However, possibly more critically, the reporting of therapy intervention in the ULIS-III cohort seemed to under-report rehabilitation practice and therapy supported practice for arm rehabilitation. Specifically application of task practice programmes such as Constraint Induced Movement Therapy^24^ or GRASP^25^ were not reported which raises the concern that that such interventions are not being offered. Similarly, electrical stimulation was not frequently applied and could be underrepresented due to the additional need for equipment and training or the practicalities of delivery in clinical environments. However, this issue could again primarily relate to the severity of presentation and how effective electrical stimulation is perceived to be in individuals with severe paresis and spasticity.

### Recording of Physical Therapy Intervention

This study used systems (ULSTR and Leg-TS) for recording physical therapy interventions in clinical practice settings (in cohort studies) for focal spasticity management in the arm and leg. These formally developed systems have been refined and developed further than other similar classification systems, for example that by Donaldson,^28^ MacFarlane et al^29^ and De Wit et al.^30^ However the complexity of constructing and developing treatment schedules^6,7,27,31,32^ should not be underestimated as emphasised in the work of DeJong et al.^33^ and emphasised by Jette in a commentary on the conclusions drawn from the work.^34^ The ULSTR and Leg-TS have taken this approach further in general and specifically in the context of rehabilitation for those with spasticity. Both tools are available for application in recording therapy intervention in practice and downloadable from the King’s College London, Department of Palliative Care, Policy and Rehabilitation:

ULSTR: https://www.kcl.ac.uk/nmpc/assets/rehab/upper-limb-physical-treatment-recording-schedule-v9-2.pdf

Leg-TS: https://www.kcl.ac.uk/nmpc/assets/rehab/leg-ts.pdf

Collecting detailed treatment intervention data in open-label studies (or routine practice) is difficult and liable to lead to missing data, as is likely to have occurred in the data sets included in this meta-analysis. Using tools such as ULSTR and Leg-TS will improve data recording and capture as demonstrated in this study, but do not entirely remove the challenges. However, linking treatment interventions and categorising them, enable a fuller understanding of these complex treatments, with potential to improve understanding and enable development of better treatment options or programmes.

### Therapy Intensity

The purpose of this meta-analysis was to use the pre-defined goal framework for spasticity management to identify what types and proportions of physical treatment intervention were provided according to goal category in the arm and the leg in people following an acquired brain injury (including stroke) receiving spasticity treatment. We did not attempt to address intensity of practice for task-practice interventions or dosage of other interventions in this study. In both the ULIS III^11,35^ and Abolish^26,27^ studies work is ongoing to look at therapy dosage in arm and leg spasticity treatment cohorts. However, intensity of rehabilitation practice and consistency with maintenance interventions require comment and are emphasised as key components in rehabilitation programmes.^36-38^ Methods and processes to increase the intensity of therapy^24,25,39^ and self-rehabilitation or maintenance need to be further considered for individuals receiving spasticity management as part of rehabilitation or long-term management programmes. At present for task-practice in the arm, no appropriate interventions are being provided (or at least recorded), which is concerning, let alone considerations of intensity or dosage.

### Strengths and Limitations

The authors recognise the following limitations to this study. Recording of rehabilitation intervention using the ULSTR and Leg-TS is reliant on report by patients and their carers. Inaccuracy in this reporting may occur and possibly inaccuracy in the treatment interventions recorded. In development of the ULSTR and Leg-TS this issue was considered, and limited inaccuracy found, but nevertheless, as with any data capture, some inaccuracy in reporting will occur. The use of the ULSTR and Leg-TS may also constrain the treatment selection to those treatments already included in these tools. However, in both cases, the option to add additional treatments options is provided and this was not used in these studies.

The utilisation of goals set using the Gaseous and Goal Attainment Scaling – Legs approach may not have been used in some cases to help direct or plan treatment. Identified goals, which are priorities for patients and carers, provide a clear starting point for the targeting of treatment intervention and may be used in this way to achieve the most important outcomes to those with spasticity. This issue may therefore have the implication that by goal setting we are also influencing the treatment provided in these studies.

## Conclusion and Future Research

In conclusion, commonalities in goal categorisation were found in arm and leg spasticity. Differences in the proportion of goals set do occur. Importantly, in these cohorts’ task-practice interventions (and to a lesser extent strength training and electrical stimulation) to improve active function (walking and transferring) were applied to rehabilitation for leg spasticity but were not frequently applied in arm rehabilitation (eg, object use, incorporation in activities of daily living task practice) for those with spasticity.

It is suggested that improved treatment planning may result in greater and faster treatment goal attainment and better outcome for stroke survivors and others following acquired brain injury. With this in mind we are in the process of developing a treatment algorithm and planning application (App) called Direct-Rehab to address this issue using the analysis presented here.
